# Phytochemical Modulation of MiRNAs in Colorectal Cancer

**DOI:** 10.3390/medicines6020048

**Published:** 2019-04-05

**Authors:** Aravinda Ganapathy, Uthayashanker Ezekiel

**Affiliations:** Department of Clinical Health Sciences, Doisy College of Health Sciences, Saint Louis University, St. Louis, MO 63104, USA; Aravindaganapathy@gmail.com

**Keywords:** colorectal cancer, phytochemicals, micro RNAs

## Abstract

Colorectal cancer (CRC) is one of the leading causes of death in the United States. Chemotherapy and radiotherapy are some of the most commonly used treatments, but are often associated with severe side effects, and are not entirely curative. It is therefore important to consider other preventative treatment options. Phytochemicals are naturally occurring bioactive compounds which have been shown to play a role in cancer prevention and treatment, especially in regards to a person’s lifestyle and diet. Recent evidence has shown that phytochemicals may exert their chemopreventative effects by targeting micro RNAs (miRNAs), which regulate the downstream expression of tumor suppressors and oncogenes. MiRNAs are small, endogenous, noncoding RNAs that regulate several biological processes through post-translational regulation. The dysregulation of miRNA expression has been shown to be associated with colorectal cancer. In this review, we will summarize and discuss several phytochemicals, which have been shown to exert chemopreventative effects in colorectal cancer by the modulation of miRNA expression.

## 1. Colorectal Cancer and Its Preventability

Cancers afflicting the colon and/or the rectum are collectively referred to as colorectal cancers (CRCs). CRC is the third most commonly diagnosed cancer in the United States and the third leading cause of cancer-related deaths in the USA [1]. Mehta et al. suggested that the high prevalence of colon cancer in the US may be linked to the Western diet: A diet rich in red and processed meat, refined starches, sugar, and trans fats, but poor in fruits, vegetables, fibers, and omega 3 fatty acids [2]. Increased red and processed meat consumption has been associated with a significantly increased risk of CRC incidence [3]. It stands to reason then, that a modification of the Western diet could potentially lead to chemopreventative or protective effects: A potential change being the increased consumption of plant-based foods such as fruits and vegetables [4]. An early hypothesis supporting plant-based diets was that the high insoluble fiber content offered protection against colorectal cancers. This postulation was first proposed by Burkitt, and supported by statistics indicating that certain African populations eating high fiber diets were found to have lower incidences of CRC as compared to populations in the United States [5]. However, studies attempting to link fiber consumption to colon cancer are weakly supportive at best—many fail to show any association at all [6,7]. Another source of interest regarding this area was the low incidence rate of CRC in India; the rates for both males and females are amongst the lowest in the world [8]. Epidemiologists attributed this difference to environmental factors such as diet; the Indian diet is far lower in processed foods and far higher in fruits and vegetables [9]. The Indian diet is also rich in spices, which contain many phytochemicals—including some that have been linked to the suppression of tumor initiation and promotion [10]. Epidemiological studies have linked the Indian spice-rich diet to reduced rates of multiple cancers, such as cancers of the colon and prostate [10]. Interestingly, data collected from cancer registries have shown that CRC incidence is lowest in Indians living in India, and higher for Indians who have migrated to areas such as the United Kingdom [11,12]. 

Interest regarding phytochemicals has accelerated recently due to the need for a safer and more effective chemoprevention agent [13]. Currently, chemotherapeutic drugs such as oxaliplatin or irinotecan are used against colon cancer, but these drugs have major side effects due to non-selectivity [14]. Thus, phytochemicals are gaining acceptance as potential chemotherapeutics, and their anti-cancer effects are being further examined. Furthermore, since phytochemicals may be a part of daily dietary habits, they may not only be utilized as part of a chemotherapeutic drug cocktail, but perhaps as a preventative nutrient when incorporated into the diet.

Alternative methods of treatment are important especially because of emerging chemoresistance to existing therapeutics. Novel treatments can inhibit tumorigenesis by different mechanisms, which may overcome current chemoresistance. Therefore, it is pertinent to investigate different molecular mechanisms of anti-tumorigenic therapeutics. A relatively recent area of interest focuses on epigenetics. Epigenetics focuses on alterations in the regulation of gene expression that does not involve a change in the specific DNA sequence of the cell [15]. Epigenetic changes include any changes in the expression of genes occurring as a result of modified DNA. One of the major methods of epigenetic modification is by DNA methylation; methylation of CpG islands near a promoter can silence the gene and dysregulate expression levels. Another method of epigenetic modification is by post-translational histone modifications such as acetylation, methylation, phosphorylation, ubiquitylation, or sumoylation. Furthermore, a large amount of focus has been recently dedicated to epigenetic modification by non-coding RNAs such as micro RNAs (miRNAs). Expression levels of miRNAs are associated with the regulation of genes involved in various cancer formations. Novel therapeutics, which may regulate the aberrant expression of these miRNAs are of interest since it offers an alternative method of treatment. Various phytochemicals have been shown to modulate epigenetic mechanisms leading to decreased tumorigenesis and aberrant cancer cell proliferation by regulating pathways linked to angiogenesis, invasion, and cell survival.

## 2. Phytochemicals and MiRNAs

### 2.1. MiRNA Processing

Phytochemicals are compounds that are derived from plant sources. Several thousand phytochemicals have been discovered, and a growing body of research has identified various molecular targets of these compounds [16]. One suggested mechanism by which phytochemicals may indirectly regulate these molecular targets is through the regulation of micro RNAs (miRNAs). MiRNAs are endogenous, small, non-coding RNA molecules that can regulate gene expression post-transcriptionally. They bind to the 3′-untranslated regions (UTRs) of target mRNA complementarily, thus preventing the mRNA from being translated [17]. The perfect binding of miRNAs to the 3′-UTR of mRNA results in transcript degradation, and the imperfect binding of miRNAs to the 3′-UTR of the transcript still results in translational inhibition [18]. The miRNA is first transcribed by RNA polymerase II into primary miRNA, and then cleaved to pre-miRNA by Drosha—an RNase III enzyme–and converted to the mature miRNA by Dicer–another RNase III enzyme [19]. This mature miRNA then associates with the RNA-induced silencing complex (RISC) which allows it to regulate the expression of various genes [20], by binding to the 3′-UTRs. These miRNAs are involved in the regulation of various biological processes such as cell proliferation, apoptosis, and differentiation [21]; when they fail to successfully regulate cell proliferation, tumorigenesis can result due to uncontrolled cell growth. Thus, abnormal upregulation or downregulation of miRNAs have been shown to play a major role in carcinogenesis [22]. Many studies have shown that phytochemicals target various miRNAs. These miRNAs could serve as intermediaries to modulate the final molecular targets and ultimately induce chemopreventative or chemoprotective effects. For example, a phytochemical could have a chemopreventative effect by downregulating a miRNA that silences a tumor suppressor gene. Alternatively, it could also have a chemopreventative effect by upregulating a miRNA that silences a proto-oncogene. A miRNA that targets a tumor suppressor gene is termed an oncomiR, and a miRNA that targets an oncogene is termed a tumor suppressor miRNA (tsmiR) [15]. 

Gene expression is regulated by epigenetic factors such as the DNA promoter methylation [15]. Aberrant hypermethylation of miRNA gene promoters can occur when they are located in or near CpG islands, which can lead to epigenetic silencing and subsequent miRNA downregulation in cancer. Inhibition of DNA methyltransferases can lead to hypomethylation of CpG island promoter, thereby upregulating miRNA. Several phytochemicals have shown that they exert their anticancer effects through the modulation of certain microRNAs [15], affecting various signaling pathways [20]. This could be a major mechanism by which phytochemicals exert their chemopreventative effects. In this review, we are focusing on recent publications focusing on phytochemicals’ anticancer effects against CRC mediated by miRNAs.

### 2.2. Curcumin

Curcumin is a yellow polyphenolic flavonoid found in turmeric, a popular spice in Indian and Asian cooking. The compound is isolated from the plant *Curcuma longa*. In the East, traditional medicine such as Ayurveda in India uses turmeric or curcumin as a treatment for a variety of disease states and conditions, such as to treat wounds or inflammation. Studies have backed some of these claims, showing curcumin to possess anti-inflammatory properties [23]. As Virchow hypothesized back in 1983, inflammatory cells are more susceptible to tumorigenesis than are healthy, normal cells [24]. This anti-inflammatory property could be a mechanism by which curcumin’s anti-tumorigenic properties are observed. Additionally, curcumin has been found to regulate the activity of several miRNAs. For example, it has been shown to induce the ROS-mediated downregulation of miR-17-5p, miR-20a, and miR-27a, which target zinc finger and BTB domain containing proteins ZBTB4 and ZBTB10 [25]. Curcumin increased the level of reactive oxygen species (ROS) within the cell and simultaneously triggered the downregulation of miR-17-5p, miR-20a, and miR-27a. The study also discovered that in the presence of glutathione (GSH), a ROS inhibitor, the expression levels of the miRNAs were rescued, demonstrating that ROS are necessary for the downregulation of miRNA-17-5p, miR-20a, and miR-27a [25]. ZBTB4 and ZBTB10 suppress specificity (Sp) proteins Sp1, Sp3, and Sp4 [25]. These Sp proteins play proto-oncogenic roles such as preventing apoptosis and allowing for cell proliferation, meaning that the downregulation of these specific miRNAs has an overall effect of inhibiting the growth of colon cancer [25]. Another study found that curcumin downregulated miR-21 in colorectal cancer cells, which in turn upregulated the tumor suppressor gene programmed cell death 4 (PDCD4) [26]. PDCD4 inhibits protein synthesis by interacting with the eukaryotic initiation factor 4A and is also known to inhibit the transformation mediated by the transcription factor activator protein-1 (AP-1) [27]. 

Curcumin has also been shown to inhibit the canonical Wnt/β-catenin pathway via the downregulation of miRNA-130a in SW480 CRC cells [28]. The Wnt/β-catenin pathway is involved in cell growth and proliferation when active, and the accumulation of β-catenin is often a hallmark of cancer cells [29]. When SW480 cells were treated with curcumin, cell proliferation was inhibited, and β-catenin levels were reduced. Furthermore, Dou et al. showed that when miR-130a was overexpressed in these curcumin-treated SW480 cells, cell proliferation and β-catenin levels were restored, demonstrating that miR-130a was responsible for these changes [28]. Loss of β-catenin accumulation resulted in the target gene transcription factor 4 (TCF4) downregulation. Naked cuticle 2 (Nkd2) is a negative regulator of the Wnt pathway, which was shown to be upregulated as well by the curcumin treatment. Therefore, the curcumin-mediated modulation of miRNA-130a led to the inhibition of cell proliferation in SW480 cells [28]. Curcumin’s anti-tumorigenic properties, therefore, proceed through the modulation of multiple molecular pathways such as the PDCD4 pathway, the Wnt/β-catenin pathway, and the ZBTB4 and ZBTB10 pathways. 

### 2.3. Difluorinated Curcumin 

Difluorinated Curcumin (CDF) is a fluoro synthetic analog of curcumin, which has been shown to be more potent in inhibiting cell growth and inducing cell death than curcumin in colon cancer cells [30]. Recent data suggest that CDF’s anti-tumor activity is mediated through miRNAs. Specifically, CDF upregulates miR-34a and miR-34c, both of which are normally downregulated in colon cancer [31]. Roy et al. suggested that the demethylation of promoter regions for miR-34a and miR-34c by CDF were responsible for the upregulation of activity [31]. The upregulation of these miRNAs leads to the downregulation of their target gene, Notch-1, an oncogene that is often upregulated in colon cancer [31]. In SW480 and HT-29 cells, the knockdown of Notch-1 significantly inhibited cell proliferation and colony formation, inducing apoptosis and cell cycle arrest [32]. CDF also downregulated miR-21, which was shown to target the tumor suppressor gene phosphate and tensin homolog (PTEN) [33]. The tumor suppressor protein PTEN can inhibit cell growth and proliferation by regulating the activation of class I PI 3-Kinase enzymes and also by modulating the Akt axis [33,34]. In the CRC cells, PTEN is often downregulated, but the downregulation of miR-21 was shown to restore expression of PTEN [33]. CDF has shown a greater bioavailability in mice compared to curcumin, suggesting potentially better therapeutic outcomes on the treatment of human cancers [35]. 

### 2.4. RL197

RL197 is a synthetic analog of curcumin which incorporates an oxopiperidine moiety [25]. Similar to curcumin, this compound has been shown to act against colon cancer cells through the ZBTB4 and ZBTB10 pathways [25]. RL197 induces ROS production in CRC cells and subsequently the ROS-mediated downregulation of miR-17-5p, miR-20a, and miR-27a. These miRNAs normally target genes ZBTB4 and ZBTB10 and inhibit their expression. The ROS-mediated downregulation of miR-17-5p, miR-20a, and miR-27a leads to the upregulation of ZBTB4 and ZBTB10, which in turn leads to the repression of Sp proteins Sp1, Sp3, and Sp4 [25]. As discussed previously, Sp proteins play a role in cell growth and survival, so the inhibition of Sp protein expression is anti-tumorigenic. The repression of Sp proteins and upregulation of ZBTB4 and ZBTB10 was attenuated by treatment with the antioxidant GSH, indicating this response was indeed mediated by ROS [25]. In RKO and SW480 cell lines, curcumin’s IC_50_ value was found to be fourteen-fold higher than RL197, suggesting that RL197’s anti-proliferative and anti-tumorigenic effects may be significantly more potent than curcumin’s. [25].

### 2.5. Resveratrol

Resveratrol is a polyphenolic stilbene found in a variety of natural sources including grapes, boiled peanuts, and herbs [36,37]. It is produced by some plants in response to environmental stimuli that induce stress or damage. Studies have shown that resveratrol has anti-cancer properties against many cancers by means of its ability to inhibit cell proliferation and induce apoptosis [38]. A key pathway by which resveratrol exerts its anti-tumor effects involves miR-96 in colon cancer cells. The target gene of miR-96 is oncogene KRAS. KRAS can trigger extracellular regulated kinase (Erk) and Akt signaling, which are associated with cell survival and proliferation, as well as the deregulation of cell shape, adhesion, and migration [39]. Resveratrol was found to upregulate miR-96 in a genetically engineered mouse model for sporadic CRC, which caused the downregulation of KRAS, an oncogene associated with tumor aggressiveness and chemoresistance [40]. Additionally, a recent study found that resveratrol altered expression in 104 out of 609 miRNAs deregulated in colon cancer–the putative targets of most being identified as related to inflammatory activity [41]. Two specific major targets include the upregulation of miR-101b and miR-455, which in turn led to decreased levels of IL-6 and TNF-α; these are pro-inflammatory proteins known to be promoters of colon cancer [41,42]. Another identified function of resveratrol is its ability to upregulate miR-663, a tsmiR that targets TGFβ1 transcripts in SW480 cells. [43]. TGFβ1 behaves as a tumor promoter in late-stage tumorigenesis by increasing angiogenesis, metastasis, and epithelial-mesenchymal-transition (EMT) [44], therefore, the downregulation of TGFβ1 led to the inhibition of cancer cell proliferation. Finally, resveratrol has been shown to increase miR-34a levels in DLD-1 and SW480 cells [45]. MiR-34a was found to target the transcription factor gene E2F3, which in turn inhibited Sirt1 expression. To demonstrate that the target of miR-34a was E2F3 and not Sirt1, Kumazaki et al. showed that the 3′-UTR binding of miR-34a to E2F3 was shown to reduce its expression level, while the 3′-UTR binding to Sirt1 was not shown to have any direct effect on Sirt1 expression level [45]. Furthermore, the gene silencing of E2F3 exhibited a marked downregulation of Sirt1, demonstrating that Sirt1 is a downstream target of E2F3 [45]. This E2F3 and Sirt1 inhibition led to the resveratrol-mediated growth inhibition and apoptotic cell death in CRC cells, contributing to resveratrol’s potential as a chemopreventative agent [45].

### 2.6. Grape Seed Extract

Grape seed extract (GSE) is formed as a byproduct of commercial grape juice and winemaking processes. The major fraction of GSE is proanthocyanidins, which are flavonoids composed of naturally occurring dimers, trimers, and other oligomers of catechin and epicatechin found in high quantities in grape seeds [46]. Grape seed extract is also the major type of polyphenol in red wine. GSE has been linked to preventative effects in cases of skin, breast, prostate, head and neck, lung, and colon cancers, likely due to its anti-oxidant and anti-inflammatory properties [47]. More specifically, the miRNA array showed the upregulation of miR-19a, miR-20a, and let-7a as well as the downregulation of miR-103, miR-135b, miR-148a, miR-196a, and miR-205 in mouse colonic mucosa with tumors after a long-term dietary feeding of GSE. GSE inhibited NF-κB activation and caused a significant reduction in colon tumor size in a dose-dependent manner [48]. High levels of NF-κB and HIF leads to the inflammatory response by enhancing the expression of pro-inflammatory cytokines [49]. According to Derry et al., the upregulation of miR-19a reportedly targets NF-κB, and miR-20a targets the HIF-1α pathway and its downstream target vascular endothelial growth factor (VEGF) [48]. VEGF regulates angiogenesis, which is associated with tumor metastasis and survival [50]. MiR-205, which was found to be downregulated by GSE, targets VEGF as well and is also known to interact with both MAPK and NOTCH pathways [48]. MiR-135b targets APC, which is a regulator of β-catenin; thus, the downregulation of miR-135b led to an increase in APC levels, which resulted in a degradation of β-catenin levels [48,51]. Let-7a was shown to be increased by GSE; let-7a is implicated in inhibiting the expression of the c-myc oncogene and is also known to inhibit the MAPK pathway [48,52]. Decreased miR-103 levels were also seen after the GSE treatment, which targets oncogenic KRAS signaling [48]. The nuclear NF-κB pathway is a key pro-inflammatory signaling pathway that has been linked to tumor progression [53]. The downregulation of NF-κB, in turn, causes the downregulation of downstream targets such as COX-2, iNOS, and VEGF, all of which are inflammatory markers raised in colon cancer [48]. The regulation of multiple miRNA targets, therefore, play a role in GSE’s anti-tumorigenic and anti-inflammatory properties; common oncogenic pathways such as NF-κB and β-catenin are downregulated in colonic tissue of GSE fed-mice, resulting in decreased tumorigenesis. Since these results were discovered after a long-term dietary feeding of GSE, this study suggests GSE as a potential dietary supplement in the prevention of colorectal cancer. 

### 2.7. Baccharin and Drupanin

Baccharin and drupanin are phenolic acids derived from the cinnamic acid, extracted from propolis. Propolis is a sticky red/brown resin substance used by honeybees to seal honeycombs. Artepillin C is another propolis extract that has been reported to inhibit tumorigenesis in colon cancer cell lines by induction of apoptosis [54]. Baccharin and drupanin have been found to inhibit colon cancer growth individually and have demonstrated an enhanced synergistic effect by supporting both the extrinsic and intrinsic apoptosis pathways [55]. The two cinnamic acid derivatives were found to increase the expression of miR-143 in DLD-1 cells, which downregulated the target gene Erk5 and its downstream target protein c-Myc [55]. C-Myc has been known to regulate expression levels of genes controlling cellular proliferation and upregulation can induce unrestricted growth and proliferation [56]. These data suggest that the supplemental feeding of the propolis-derivatives baccharin and drupanin could be significant in cancer prevention, especially due to a potential synergistic effect.

### 2.8. Methyl 2-cyano-3,11-dioxo-18β-olean-1,12-dien-30-oate

Methyl 2-cyano-3, 11-dioxo-18β-olean-1, 12-dien-30-oate (CDODA-Me) is a synthetic derivative of glycyrrhetinic acid, a triterpenoid phytochemical extracted from licorice [57]. Treatment of RKO and SW480 CRC cells with CDODA-Me was found to decrease expression of miR-27a, resulting in increased levels of its target mRNAs ZBTB10 and Myelin transcription factor 1 (Myt1) [57]. As discussed earlier, ZBTB10 acts to repress levels of specificity proteins Sp1, Sp3, and Sp4 [25]. Myt1 kinase is a cell cycle regulator which can act to prevent progression to the G2/M phase [58]. Thus, the upregulation of Myt1 allows repression of cell cycle progression and subsequently cell growth termination. These studies suggest potential chemotherapeutic effects for CDODA-Me either by dietary supplement or in treatment. 

### 2.9. Sulforaphane

Sulforaphane is an isothiocyanate derivative found in some plants of the cabbage family, including kale, cabbage, and broccoli sprouts. It was found to be a natural potent histone deacetylase (HDAC) inhibitor [59]. Histone deacetylases catalyze the removal of an acetyl group from histones. The presence of the acetyl group serves to neutralize the normal positive charge of the histone core, thus weakening the interaction between the DNA and the histones, and also making the DNA more accessible for transcription. Thus, a histone deacetylase serves to make the DNA less accessible to transcription, and a histone deacetylase inhibitor serves to make the DNA more accessible by inhibiting the removal of the acetyl group. Sulforaphane treatment on NCM460 and NCM356 normal colonic epithelial cell lines was found to induce an alteration in expression levels of several miRNAs, such as the upregulation of tsmiRs miR-23b and miR-27b (which are part of the miR-23b cluster) and the downregulation of oncomiR miR-155, which may contribute to the chemoprotective effects of sulforaphane [60]. Epithelial-mesenchymal transition is a process which allows polarized epithelial cells to assume a mesenchymal cell phenotype and leads to metastasis and chemoresistance of CRC [61]. MiR-23b has been found to inhibit this EMT [62]. In a 2011 study, miR-23b was found to downregulate proteins FZD7 and MAP3K1, two pro-metastatic genes, in HCT116 CRC cells [63]. Another study found that the treatment of sulforaphane decreased cell density in RKO cells and inhibited tumorigenesis; one mechanism identified was by decreasing miR-21, which caused the downregulation of human telomerase reverse transcriptase (hTERT) and HDAC1 [59]. The downregulation of miR-21 leads to the upregulation of PTEN, which in turn, inhibits the Akt pathway, leading to the decreased hTERT expression [59]. Human telomerase reverse transcriptase is the catalytic subunit of telomerase, which is implicated in several cancers [64].

The miR-155 regulation may affect anti-inflammatory pathways, as it has been linked to the immune response [65]. MiR-155 has been shown to bind the 3′-UTR of Suppressor of Cytokine Signaling 1 (SOCS1), thus inhibiting its expression [66]. In another study, SOCS1 was seen to exert its tumor suppressor activity in colorectal cancer cells by reducing tumor cell invasion and inhibiting EMT [67]. MiR-155 was also found to be responsible for upregulating AKT activity by binding to the 3′-UTR of Protein Phosphatase 2 Catalytic Subunit Alpha (PPP2CA), which is a known suppressor of Akt [66]. Therefore, sulforaphane treatment decreased miR-155 expression, which likely inhibited tumorigenesis by the modulation of the Akt cascade as well. Sulforaphane, therefore, may act through the modulation of inflammatory cascades, EMT, and telomerase to exert its anti-tumorigenic activity.

### 2.10. Walnuts

Walnuts have a particularly high phenolic content with considerable amounts of syringic acid and juglone, and minor amounts of proanthocyanidins and flavonoids [68]. Walnuts have been shown to suppress colon cancer in mice models through the decreased expression of miR-1903, miR-467c, and miR-3068, as well as the increased expression of miR-297a in athymic nude mice injected subcutaneously with HT-29 CRC cells [69]. An equivalent of two human servings of walnuts per day were ground up in a food processor, mixed with corn oil and then fed to the mice. Tsoukas et al. suggested that the upregulation of miR-297a suppressed cyclooxygenase enzymes, which thus conferred an anti-inflammatory effect, contributing to walnuts’ anti-tumorigenic properties in mice [69]. The downregulation of miR-467c was also observed in walnut-fed mice. Micro RNA-467c regulates several tumor suppressor genes: FAT tumor suppressor homolog 4 (FAT4), a cadherin-like protein, fibroblast growth factor receptor 2 (FGFR2), a receptor for FGFs, nuclear receptor coactivator-3 (NCOA3), a transcriptional coactivator, and LIM domain transcription factor-4 (LMO4), a transcriptional regulator [69]. MiR-1903 was found to be downregulated as well and has several putative targets: SAP domain containing ribonucleoprotein (SNARP), which regulates transcription and DNA repair, and RNA Binding Motif Protein 25 (RBM25), which is involved in the regulation of apoptotic cell death [69]. Additionally, tumors were found to have increased levels of eicosapentaenoic acid, docosahexaenoic acid, α-linolenic acid, and total omega-3 acids, with tumor size negatively related to the percentage of omega-3 acid composition of the tumor [69]. The modulation of multiple molecular miRNA targets appears to inhibit tumor growth and proliferation by interfering with the inflammatory response, as well as cell migration, transcription, and aberrant DNA repair, pointing to the significance of walnuts as potential chemotherapeutic or chemopreventative agents. 

### 2.11. Extra Virgin Olive Oil

Extra virgin olive oil (EVOO) contains a high concentration of phenolic compounds such as hydroxytyrosol and oleuropein [70]. EVOO has been shown to inhibit human colon cancer. Treatment of Caco-2 CRC cells with EVOO caused the downregulation of miR-23a and miR-301a, which were predicted to target type 1 cannabinoid receptor (CB_1_) by Di Francesco et al. [71]. EVOO did cause an increase in the expression of CNR1, a gene encoding CB_1_. CB_1_ is a tumor suppressor which protects against colonic inflammation and regulates apoptosis and cell survival [71,72]. Phenolic extracts of EVOO, when administered to colon cancer cells, decreased DNA methylation at the promoter CNR1, which in turn increased expression of CB_1_. This was correlated with a lower viability of the colon cancer cells, indicating that EVOO has important implications in chemoprevention [71].

### 2.12. α-Mangostin

The compound α-mangostin is a xanthone from the pericarps of the mangosteen plant. The pericarps of this plant have historically been used to treat skin infections and wounds, as well as gastrointestinal complaints. α-Mangostin was found to exhibit in vitro cytotoxicity against DLD-1 cells by triggering apoptosis [73]. The mechanism of apoptotic induction was explored and was found to be mediated by the release of endonuclease-G (endo-G) from the mitochondria. Furthermore, the activation of MAP kinases in α-mangostin-induced apoptosis was examined, and Erk5 expression levels were seen to gradually decrease after treatment. This was attributed to a significant dose-dependent increase in miR-143 levels [73], which has been shown to repress Erk5 levels [74]. α-Mangostin, therefore, exerts its anti-tumorigenic effects by the induction of apoptosis, and the repression of MAPK and Erk5 pathways, making it a potentially attractive chemotherapeutic agent or dietary supplement. 

### 2.13. Boswellic Acid (AKBA) 

Boswellic acids make up the gum resin derived from the plant *Boswellia serrata*. Like many of the other phytochemicals discussed in this review, boswellic acids have traditionally been used to treat inflammation and wounds as an antimicrobial and anti-inflammatory [75]. Acetyl-11-keto-β-boswellic acid (AKBA) is one of the active components present in boswellic acids and is a pentacyclic triterpene. It has been found to exert anticancer effects in CRC cell lines by the upregulation of the let-7 and miR-200 families [76]. Both of these miRNA families have been shown to be involved in regulating the epithelial-mesenchymal-transition (EMT), which is involved in the metastasis of cancer cells [77,78]. The downstream expression of target genes was then explored in three colorectal cancer cell lines: HCT116, HT-29, and SW620. E-cadherin levels were significantly increased in HCT116 and HT-29 cells and trended towards increasing in SW620 cells as well. Levels of CDK6 were decreased in all three cell lines tested. Vimentin protein levels were found to decrease significantly in SW620 cells. Vimentin and E-cadherin are both proteins that are involved in the EMT; vimentin being a constituent of intermediate filament proteins which is highly expressed by mesenchymal cells, and thus correlates with accelerated tumor growth and invasion, while E-cadherin is a transmembrane glycoprotein involved in cell-cell adhesion which acts to limit EMT and metastasis [79,80]. The same trend held up in vivo when HCT116 cells were injected into the cecum of nude mice, and AKBA was administered by gavage [76]. Thus, not only does AKBA inhibit cell proliferation in a dose-dependent manner, but also inhibits cell migration and interferes with EMT in CRC cells. Inhibition of the cell migration and EMT plays a direct role in interfering with metastasis and the transformation of cells, which suggests AKBA may be useful as a dietary supplement in protecting against aggressive cancers. 

### 2.14. Plum Polyphenols

The anti-inflammatory and anti-tumorigenic properties of plum polyphenols such as chlorogenic acid and neochlorogenic acid, both phenolic acids, have been investigated in azoxymethane-treated Sprague-Dawley rats. The plum beverage treatment was shown to increase the expression levels of miR-143, which regulates the Akt/mTOR pathway [81]. Accordingly, Akt, pAkt, and mTOR expression levels were decreased, suggesting that the mechanism of plum polyphenols’ anticancer activity is by repression of the Akt/mTOR axis through miR-143 upregulation [81]. As discussed previously, Akt is associated with cell survival and proliferation, so repression of the pathway inhibits aberrant cell survival, and suggests chemopreventative potential against colon carcinogenesis. 

### 2.15. Spica Prunellae

Spica prunellae is the spike of the herb *Prunella vulgaris*. Spica prunellae has been used in traditional Chinese Medicine to remedy various illnesses [82]. The phytochemical has been shown to possess anticancer activity by promoting apoptosis and inhibiting angiogenesis of CRC cells in vivo [83]. One study found that Spica prunellae upregulated miR-34a in HCT-8 human colon carcinoma cells, which triggered the subsequent downregulation of target genes Notch1, Notch2, and Bcl-2, leading to the inhibition of cell viability, and induced apoptosis in HCT-8 cells [82]. The inhibition of angiogenesis is especially important in preventing metastasis and increased apoptosis by the regulation of Notch and Bcl-2 signaling suggests that Spica prunellae could be incorporated into chemotherapeutic regimens, or perhaps even simply into long term diets as a chemoprotective agent. 

### 2.16. Ellagitannins

Ellagitannins are polyphenol tannins abundant in pomegranates, walnuts, and certain berries, and are metabolized by healthy gut microbiota to form dibenzopyran-6-one derivatives referred to as Urolithins [84]. The exposure of Caco-2 CRC cells to Urolithin-D, Urolithin-C, Urolithin-A, Isourolithin-A, and Urolithin-B triggered the downregulation of miR-224. The exposure of HT-29 CRC cells to Ellagic acid, Urolithin-D, Urolithin-C, Urolithin-A, Isourolithin-A, and Urolithin-B triggered the upregulation of miR-215. MiR-224 is an oncomiR which downregulates p21 [85], while miR-215 is a tsmiR which indirectly increases p53 and p21 (also known as CDKN1A) by downregulating the G2 checkpoint regulator denticleless protein homolog (DTL) [86]. In both cell lines, the induction of cyclin-dependent kinase inhibitor 1A (CDKN1A) was seen [87]. CDKN1A acts as a tumor suppressor by triggering cell-cycle arrest [88]. Ellagitannins appear to exert anti-tumorigenic activity by regulation of the cell cycle as several urolithins upregulated p21 and induced cell cycle arrest. 

### 2.17. Rosemary Extract

Rosemary components such as carnosic acid and carnosol have been proposed to have anti-tumor effects [89]. Carnosol is a naturally occurring phenolic diterpene, while the carnosic acid is an abietane diterpenoid. In SW480 cells, the rosemary extract was shown to downregulate miR-15b, which was predicted to target Glucosaminyl transferase 3 (GCNT3) by in silico analysis [90]. GCNT3 has been shown to be a tumor suppressor in CRC by reducing cell growth and invasion [91]. Rosemary extracts thus help to prevent cancer cell transformation and dysplasia by induction of GCNT3.

### 2.18. Methyl Jasmonate

Methyl jasmonate is the methyl ester of jasmonic acid, which is a member of the jasmonate class of plant hormones. Methyl jasmonate has been reported to induce the apoptosis of lymphocytic leukemia cells and inhibit the proliferation of myelogenous leukemia [92]. Enhancer of zeste homolog 2 (EZH2) is a gene which has been shown to be overexpressed in a variety of cancers and assist in their proliferation [93]. In SW620 cells, methyl jasmonate was shown to upregulate miR-101 levels, which downregulated the expression of target gene EZH2 [94]. Methyl jasmonate, therefore, demonstrates anti-tumorigenic activity in multiple cancers, both lymphocytic leukemia as well as CRC cell lines, suggesting a broad range of potential chemotherapeutic application. 

### 2.19. American Ginseng

American ginseng (AG) is a herb native to North America, in which the major phytochemical constituents are triterpenoid saponins or ginsenosides. A hexane fraction of AG (HAG) has been shown to be a potent anti-oxidant and anti-tumorigenic agent in CRC cells [95]. HAG was found to significantly upregulate the miR-29b expression in HCT116, DLD-1, and LOVO cells. A corresponding reduction in the MMP-2 activity was discovered, and MMP-2 was confirmed as a target gene of miR-29b. When miR-29b was silenced, the MMP-2 gene expression was restored [96]. MMP-2 is a key regulator of cell migration; HAG suppressed the migration of cancer cells by nearly 7-fold [96], contributing to its effectiveness as an anti-tumor agent.

## 3. Conclusions

**Figure 1:**
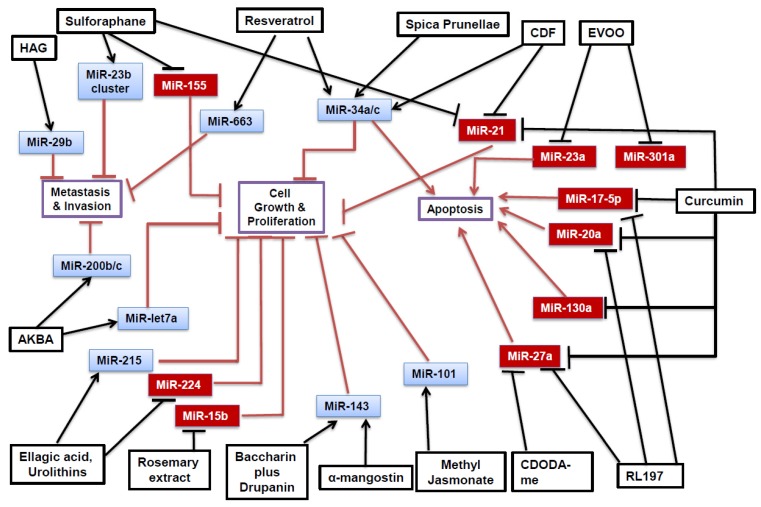
In vitro effect of phytochemicals against colorectal cancer by modulation of micro-RNA. Black arrows indicate upregulation while blunted black bars indicate downregulation of selected miRNA by phytochemicals. Overall phytochemical modulation of miRNAs exerts anti-tumorigenic effects either by inhibition of metastasis and invasion, inhibition of cell growth and proliferation, or induction of apoptosis. OncomiRs are shown in red boxes and are downregulated by phytochemicals, while tsmiRs are shown in blue boxes and are upregulated by phytochemicals.

Phytochemicals have a great potential as novel anti-tumorigenic therapeutics. Several phytochemicals have been identified which can inhibit the growth of colon cancer by the epigenetic modulation of miRNAs. Furthermore, several oncomiRs and tsmiRs have been identified which mediate the tumorigenic cascade for colorectal cancers. Common miRNAs involved in the colorectal carcinogenesis include oncomiRs miR-21 and miR-27a, and tsmiR miR-34a; many anti-tumorigenic pathways appear to rely on these miRNAs, as displayed in Figure 1. These miRNAs appear to regulate various effectors and pathways within the cell. The phytochemical modulation in vitro appeared to most often affect pathways modulating cell growth and proliferation, metastasis, and apoptosis, as illustrated in Table 1. In experiments performed in vivo, many of the miRNAs appeared to affect inflammatory pathways, as shown in Table 2. Phytochemicals’ anti-carcinogenic effect is mediated by either downregulating proteins involved in metastasis or cell growth (such as MMP-2 or Akt), or by upregulating proteins involved in the apoptotic cascade. 

Phytochemicals could potentially be used in combination therapies with current chemotherapeutic treatments. For example, if an aberrant dysregulation of the Wnt pathway were identified in a certain colorectal tumor, curcumin in combination might be an appropriate treatment, as it was found to downregulate the Wnt pathway in SW480 cells. Likewise, combination therapeutics can take advantage of microarray technology to identify aberrant pathways and identify appropriate phytochemicals to be used in treatment. Cancer is often a result of multiple mutations within a cell. Therefore, it is possible that the mutations make cancer resistant to one type of drug or another, which is why combination therapies have become popular. Targeting cancer cells by multiple anti-tumorigenic therapeutics allows a multi-faceted approach to inhibiting growth and proliferation of cancer. Use of phytochemicals alone or in combination for treatment can help to regulate aberrant molecular pathways in cancer cells, such as the NF-κβ and the Akt/mTOR pathway. 

Even though several in vitro and in vivo studies have shown phytochemicals to have anticancer effects, their use has been limited by low bioavailability [97,98,99]. One well studied compound in regards to bioavailability is the turmeric compound curcumin [97]. Curcumin possesses poor bioavailability and low stability in the bloodstream, but its effect in vivo has been attributed to its metabolites and degradative products [97,100]. Shen et al. demonstrated that the degradative products showed higher superoxide scavenging activity and stronger inhibitory activity against Aβ fibril formation than curcumin [100]. In addition, studies have demonstrated that curcumin stability and bioavailability can be increased by polymeric nanoparticle formation and liposome emulsion methods [101]. Its bioavailability also can be increased when curcumin was taken together with piperine, a phytochemical from black pepper [102]. The concomitant administration of piperine with curcumin was shown to increase bioavailability by 2000%. Thus, the effect observed by a phytochemical may be due to metabolite or degradative products. Furthermore, the bioavailability of phytochemicals can be improved by techniques such as encapsulation and conjugation. 

Epigenetic dysregulation can occur in an early stage of cancer development and contribute to tumor progression [103]. If these epigenetic changes are reversed by phytochemicals, they could potentially prevent the initiating step of cancer development. Therefore, phytochemicals that alter epigenetic changes are promising new agents for cancer prevention. Another advantage of using natural compounds such as phytochemicals is that they can aid in mitigating the severe side effects prevalent in chemotherapy and radiation therapy. With the significant incidence of colon cancer in the US, plant-based compounds offer a new avenue with which to examine the treatment of CRC. Furthermore, many of these phytochemicals can be incorporated easily into a diet to offer a preventative effect against colon cancer and other cancers in the long term. 

Recently, Tabung et al. reported that there is a link between an inflammation-inducing diet and the risk of developing colon cancer [104]. In addition, chronic inflammatory diseases such as ulcerative colitis and Crohn’s disease have been associated with colon cancer development as well [105]. Therefore, one could expect that reducing inflammation will lead to reduced cancer risk. Studies have shown that the use of non-steroidal anti-inflammatory drugs (NSAIDs), such as aspirin and ibuprofen, have been linked to reduced cancer risk [106]. However, the long-term use of NSAIDs can lead to common side effects such as gastrointestinal damage [107]. As many in vivo experiments have demonstrated the mitigating effect of phytochemicals on inflammation, it stands to reason that the incorporation of phytochemicals into one’s diet would be an overall safer method for long term cancer prevention. 

As shown in Table 2, one of the primary mechanisms by which miRNAs exert their anticancer effect in vivo is by the inhibition of inflammation. For example, in resveratrol-treated mice the reduction in tumor size correlated with miR-101b and miR-455 upregulation, which in turn led to the downregulation of the target cytokines IL-6 and TNF-alpha (Table 2). Recently, the miRNA regulatory activity in ulcerative colitis and other inflammatory bowel disorders (IBDs) has been elucidated [108], so it’s possible that phytochemical treatment could work to alleviate some of these inflammatory symptoms as well. Individuals with ulcerative colitis are known to be at higher risk of developing colorectal cancer, so this supports previously discussed phytochemical anti-tumorigenic activity. Though IBDs are one mechanism of CRC onset, they are not the only triggering factor. 

Several mechanisms of tumor inhibition by miRNA modulation have been reviewed here. The phytochemicals in combination can mediate anti-cancer effects synergistically by modulating miRNAs. This knowledge will allow innovative treatments for CRC to be identified, which can offer preventative effects, complement traditional chemotherapy, or become treatments of their own.

## Figures and Tables

**Table 1:** In vitro effect of phytochemicals against colorectal cancer by miRNA regulation.

Phytochemical	Cell Line	MiRNA Affected	Target(s) and Effect	Reference
Curcumin	RKO, SW480	miR-17-5p↓ miR-20a↓ miR-27a↓	ZBTB10↑, ZBTB4↑ Sp1↓, Sp3↓, Sp4↓	[25]
	RKO, HCT116	miR-21↓	PDCD4↑	[26]
	SW480	miR-130a↓	Wnt↓, B-catenin↓, Nkd2↑	[28]
CDF	HCT-116, SW620, HT-29	miR-21↓	PTEN↑	[33]
	HCT-116, SW620	miR-34a↑ miR-34c↑	Notch-1↓	[31]
RL197	RKO, SW480	miR-17-5p↓ miR-20a↓ miR-27a↓	ZBTB10↑, ZBTB4↑ Sp1↓, Sp3↓, Sp4↓	[25]
Resveratrol	DLD-1, SW480	miR-34a↑	E2F3↓, Sirt1↓	[45]
	SW480	miR-663↑	TGFβ1↓	[43]
Baccharin + Drupanin	DLD-1	miR-143↑	MAPK/Erk5↓ C-myc↓	[55]
CDODA-Me	RKO, SW480	miR-27a↓	ZBTB10↑, Myt1↑ Sp1↓, Sp3↓, Sp4↓	[57]
Sulforaphane	RKO	miR-21↓	hTERT↓, HDAC1↓	[59]
	NCM460, NCM356	miR-23b↑ miR-27b↑	FZD7↓ MAP3K1↓	[60] [63]
	NCM460, NCM356	miR-155↓	SOCS1↑, AKT↓	[60,66]
EVOO	Caco-2	miR-23a↓ miR-301a↓	CB_1_↑	[71]
A-mangostin	DLD-1	miR-143↑	MAPK/Erk5 ↓	[73]
AKBA	SW620, HT29, HCT116	miR-200b↑ miR-200c↑ miR-let7a↑	Vimentin↓ CDK6↓ E-cadherin↑	[76]
Spica Prunellae	HCT-8	miR-34a↑	Notch-1↓ Notch-2↓ Bcl-2↓	[82]
Ellagic Acid and Urolithins	HT-29, Caco-2	miR-215↑ miR-224↓	CDKN1A↑	[87]
Rosemary Extract	SW480	miR-15b↓	GCNT3↑	[90]
Methyl Jasmonate	SW620	miR-101↑	EZH2↓	[92]
HAG	HCT116, DLD-1, LOVO	miR-29b↑	MMP-2↓	[96]

Increase in expression levels of miRNAs and proteins are shown by an up arrow (↑) and decrease in expression levels of miRNA and protein are shown by a down arrow (↓).

**Table 2:** In vivo effect of phytochemicals against colorectal cancer by miRNA regulation.

Phytochemical	Cell Line Tested	miRNA Affected	Target(s) and Effect	Reference
Resveratrol	APC^CKO^/Kras^mut^ mice	miR-96↑	Kras↓	[40]
	Apc^Min/+^ mice	miR-101b↑ miR-455↑	IL-6↓, TNF-α↓	[41]
Grape Seed Extract	Azoxymethane (AOM)-induced colon tumors in A/J Mice	miR-19a↑ miR-20a↑ miR-103↓ miR-135b↓ miR-148a↓ miR-196a↓ miR-205↓ miR-let7a↑	NF-κB↓ β-catenin↓ pERK1/2↓ HIF-1α↓ Kras↓ VEGF↓ C-myc↓	[48]
Walnuts	HT-29 injected into mice	miR-297a↑ miR-467c↓ miR-1903↓ miR-3068↓	Cyclooxygenase enzymes↓ FAT4↑ FGFR2↑ NCOA3↑ LMO4↑ PIGR↑ SNARP↑ RBM25↑	[69]
Plum Polyphenols	AOM-induced colon tumors in Sprague-Dawley rats	miR-143↑	Akt↓, mTOR↓	[81]

Increase in expression levels of miRNAs and proteins are shown by an up arrow (↑) and decrease in expression levels of miRNA and protein are shown by a down arrow (↓).
